# The late‐annotated small ORF *LSO1* is a target gene of the iron regulon of *Saccharomyces cerevisiae*

**DOI:** 10.1002/mbo3.303

**Published:** 2015-10-08

**Authors:** Xiuxiang An, Caiguo Zhang, Robert A. Sclafani, Paul Seligman, Mingxia Huang

**Affiliations:** ^1^ Department of Biochemistry and Molecular GeneticsUniversity of Colorado School of MedicineAuroraColorado80045; ^2^ Division of HematologyDepartment of MedicineUniversity of Colorado School of MedicineAuroraColorado80045

**Keywords:** Cell cycle, iron, regulon, transcriptome, yeast

## Abstract

We have identified a new downstream target gene of the Aft1/2‐regulated iron regulon in budding yeast *Saccharomyces cerevisiae*, the *l*ate‐annotated *s*mall *o*pen reading frame *LSO1*. *LSO1* transcript is among the most highly induced from a transcriptome analysis of a *fet3‐1* mutant grown in the presence of the iron chelator bathophenanthrolinedisulfonic acid. *LSO1* has a paralog, *LSO2*, which is constitutively expressed and not affected by iron availability. In contrast, we find that the *LSO1* promoter region contains three consensus binding sites for the Aft1/2 transcription factors and that an *LSO1‐lacZ* reporter is highly induced under low‐iron conditions in a Aft1‐dependent manner. The expression patterns of the Lso1 and Lso2 proteins mirror those of their mRNAs. Both proteins are localized to the nucleus and cytoplasm, but become more cytoplasmic upon iron deprivation consistent with a role in iron transport. *LSO1* and *LSO2* appear to play overlapping roles in the cellular response to iron starvation since single *lso1* and *lso2* mutants are sensitive to iron deprivation and this sensitivity is exacerbated when both genes are deleted.

## Introduction

Iron is an essential element for life because it is used as a cofactor in many key enzymes required in diverse biological processes. On the other hand, abnormal accumulation of iron can be highly toxic to the cell due to its chemical reactivity. Therefore, cells have evolved various regulatory mechanisms to maintain a balance between iron deprivation and iron overload by controlling cellular iron uptake, intracellular iron trafficking, and utilization of iron. Iron homeostasis in the budding yeast *Saccharomyces cerevisiae* is largely regulated at the level of transcription and mRNA stability (Philpott and Protchenko 2008; Outten and Albetel 2013). Yeast cells respond to iron starvation by activating two paralogous iron‐dependent transcription factors Aft1 and Aft2, which activate transcription of over 20 genes that are collectively named the iron regulon (Yamaguchi‐Iwai et al. 1995; Rutherford et al. 2001).

Aft1 and Aft2 bind overlapping, albeit distinct target DNA sequences at their target gene promoters (Rutherford et al. 2003). Aft1 appears to play a major role in transcriptional activation of the iron regulon as *aft1Δ* cells exhibit a severe growth defect under iron‐deficient conditions, while cells lacking Aft2 do not (Yamaguchi‐Iwai et al. 1995; Rutherford et al. 2001). However, deletion of *AFT2* in *aft1Δ* mutant exacerbates the growth defect under iron‐starved conditions, suggesting functional overlap between Aft1 and Aft2 (Rutherford et al. 2001). Both nuclear localization of the Aft1 and Aft2 proteins and their occupancy at the target promoters are subjected to negative regulation by protein–protein interactions that involve Grx3, Grx4, Fra2, and Fra1 in combination with iron–sulfur clusters (ISCs) (Ojeda et al. 2006; Pujol‐Carrion et al. 2006; Kumanovics et al. 2008; Li et al. 2009, 2011; Mühlenhoff et al. 2010). A Cys‐Asp‐Cys (CDC) motif shared by Aft1 and Aft2 is essential for in vivo iron signaling, self‐dimerization, and interaction with Grx3/4 (Yamaguchi‐Iwai et al. 1995; Rutherford et al. 2005; Ueta et al. 2007). Evidences from genetic studies suggest that Aft1 and Aft2 respond to changes in cellular iron level by sensing the status of the mitochondrial ISC biogenesis (Chen 2004; Rutherford et al. 2005). Consistent with this notion, binding to ISCs via the CDC motif promotes Aft2 dimerization and weakens its DNA‐binding activity (Poor et al. 2014).

Genes in the iron regulon encode proteins that function in cell surface iron transport (*FET3*,* FTR1, FRE1*, and *FRE2*), in siderophore iron retention (*FIT1‐3* and *ARN1‐4*) (Yun et al. 2000, 2001), as well as in iron transport across the vacuolar (*FET5*,* FTH1*,* FET5*, and *SMF3*) and mitochondrial (*MRS4*) membranes (Courel et al. 2005). In addition, the iron regulon also includes two paralogous genes *CTH1* and *CTH2*, which encode mRNA‐binding proteins that facilitate degradation of mRNAs coding for iron‐requiring proteins that function in iron‐rich metabolic pathways so as to reprioritize iron utilization under iron‐deficient conditions (Puig et al. 2005, 2008; Martínez‐Pastor et al. 2013). Upon iron starvation, cells induce *FET3‐FTR1* encoding the high‐affinity multicopper oxidase–iron permease complex via Aft1‐mediated transcriptional activation to increase iron uptake at the cellular surface (Askwith et al. 1994; Stearman et al. 1996). In this study, we examined genome‐wide transcript level changes in response to iron chelation in a sensitized *fet3‐1* mutant background. At the top of the list of the genes that are highly induced by iron starvation is the *l*ate‐annotated *s*mall *o*pen reading frame *LSO1*, which has recently been shown to be induced by copper‐BPQ (2‐(6‐benzyl‐2‐pyridyl)quinazoline) treatment that induces many genes of the iron regulon (Foster et al. 2014a). For the first time, we provide evidences that *LSO1* is an authentic downstream transcriptional target of Aft1. *LSO1* has a paralog *LSO2*, the expression of which is not regulated by iron. Simultaneous removal of *LSO1* and *LSO2* exacerbates the slow growth defects of *fet3Δfet4Δ* mutants lacking both the high‐ and low‐affinity iron uptake systems. These results highlight the functional roles of these two small genes for cellular survival under iron starvation.

## Experimental Procedures

### Yeast strains, plasmids, and media

*Saccharomyces cerevisiae* strains and plasmids used in this study are listed in Table 1. Cells were grown in standard Yeast Extract Dextrose (YPD) or Synthetic Dextrose (SD) medium. For bathophenanthrolinedisulfonic acid (BPS)‐YPD plates, 100 *μ*mol/L of BPS was added. For ferrozine plates, 1 mmol/L of ferrozine was added, which has the capacity of chelating up to 333 *μ*mol/L of Fe, and 25 *μ*mol/L of Fe^2+^ was then added. Yeast strains were constructed using tetrad analysis and PCR to screen for mutant combinations.

**Table 1:** Yeast strains used in this study

Strain	Relevant genotype
DY150‐6	*MATa ade2‐1 ura3‐52 leu2‐3,112 trp1‐1 his3‐11,15 can1‐100 fet3‐1*
BY4742	*MATα his3Δ1 leu2Δ0; lys2Δ0; ura3Δ0*
AXY1422	BY4742, *lso1::HIS3*
AXY1438	BY4742, *lso2::HIS3*
AXY1443	BY4742, *lso1::HIS3 lso2::HIS3*
AXY2133	BY4742, *fet3::KanMX4 lso1::HIS3 lso2::HIS3*
AXY2136	BY4742, *fet3::KanMX4 lso1::HIS3*
AXY2138	BY4742, *fet3::KanMX4 lso2::HIS3*

Plasmid	Description
pMH1794	pRS413‐P_LSO1_‐LSO1‐3xHA
pMH1795	pRS413‐P_LSO2_‐LSO2‐3xHA
pMHx1807	pRS416‐P_LSO1_‐LacZ

### Yeast microarray analysis

For the microarray analysis, *fet3‐1* strain DY150‐6 (Askwith et al. 1994) was grown to log phase (1–2 × 10^6^/mL) in supplemented minimal SD medium (1–2 *μ*mol/L FeCl_3_) and then 100 *μ*mol/L of BPS was added, which is more than sufficient to chelate most of the iron for 18 h at 30°C. Both control and BPS cultures grew to about 4 × 10^7^ cells/mL and then were harvested. RNA was isolated by phenol extraction of whole cells (Sclafani and Fangman 1984). The experiment was done twice with one control and two BPS cultures used each time. Affymetrix yeast chips with 11 probe pairs per gene and Gene Chip software version 1.4 were used (Table S1). It is a statistical analysis based on *t*‐values and the false‐discovery rate (FDR) for the top 178 genes affected. At a FDR of 0.006, we would expect only one (0.006 × 178 = 1.068) to be wrong just by chance. Therefore, these 178 are very significant.

### Cell cycle and immunoblot analyses

Flow cytometry of yeast cells stained with propidium iodide and immunoblot of total yeast protein extracts were as described previously (Leon et al. 2008; Wu and Huang 2008). Cell sizes were determined using a Beckman–Coulter counter Multisizer III using latex beads as size standards. Budding was measured using phase‐contrast microscopy at 400× magnification. Clb5‐3XHA plasmid HA‐dR1 was transformed into strain DY150‐6 to allow detection of Clb5 by anti‐HA immunoblot (Cross and Jacobson 2000). Clb2 was detected using a rabbit polyclonal anti‐Clb2 antibody from Dr. Doug Kellogg at UC Santa Cruz (Kellogg and Murray 1995).

#### β‐galactosidase activity assay

Yeast cells were grown overnight in SC‐Ura selective liquid medium to stationary phase, diluted 1:100, and grown in fresh SC‐Ura medium to early log phase (OD_600_ ≈ 0.5). Liquid *β*‐galactosidase assays were performed on chloroform and sodium dodecyl sulfate–permeabilized cells by using the colorimetric substrate *o*‐nitro‐phenyl‐*β*‐galactopyranoside (ONPG; Sigma, St. Louis, MO) as described previously (Elledge and Davis 1989). The *P‐*value is calculated based on independent two‐sample *t*‐test.

#### Indirect immunofluorescence

Fluorescence and DIC microscopy were performed using an E800 microscope (Nikon, Chiyoda, Tokyo, Japan) with the Metamorph imaging software (Molecular Devices, Sunnyvale, CA) as described previously (An et al. 2006). Mouse monoclonal anti‐HA (12CA5) and Fluorescein isothiocyanate (FITC)‐conjugated goat‐anti‐mouse antibodies were used.

## Results

### Iron deprivation leads to G1 cell cycle arrest

**Figure 1:**
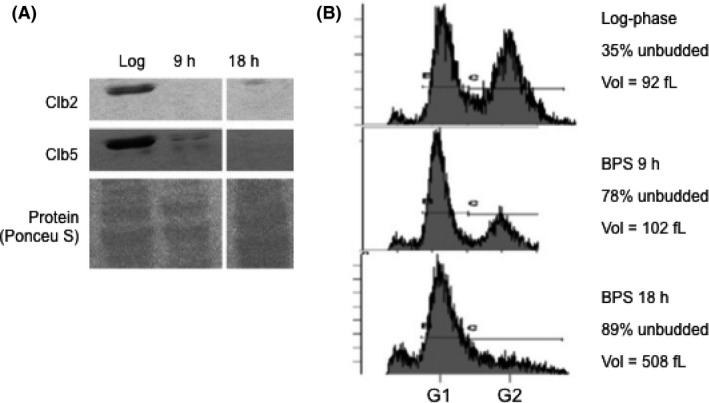
Yeast *fet3* mutant cells grown under iron deficiency accumulate in G1 phase of the cell cycle. The *fet3‐1* mutant was grown in supplemented minimal SD medium to log phase before the addition of 100 *μ*mol/L bathophenanthrolinedisulfonic acid (BPS) to the culture. Cells were harvested at 9 and 18 h post‐BPS treatment for western blot and flow cytometry analyses. (A) Protein immunoblot of S‐phase cyclins Clb2 and Clb5. (B) Flow cytometry profiles, budding index, and average cell volumes.

**Figure 2:**
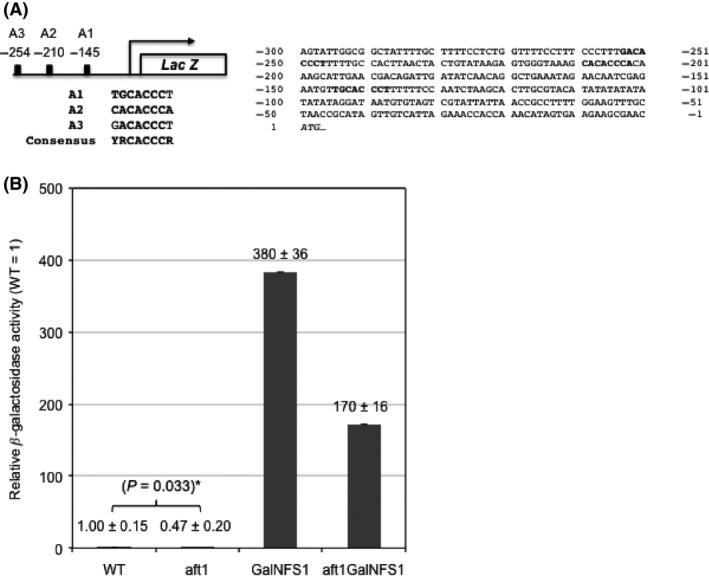
*AFT1*‐dependent activation of *LSO1* transcription in cells deficient in Fe‐S cluster biogenesis. (A) The *LSO1* upstream sequence (‐1 to ‐250 nucleotides from *LSO1* start codon) contains three sequences that resembles the consensus (YRCACCCY, Y: T/C, R: G/A) Aft1/2‐binding site, with best fit (8/8) at ‐210. The first ATG of ORF is in italics. (B) A *P*_*LSO*__*1*_ *‐LacZ* reporter plasmid containing the *LSO1* upstream sequence (‐1 to ‐476) fused the *Escherichia coli LacZ* open reading frame was introduced into wild‐type (WT), *aft1Δ*,* GalNFS1*, and *aft1Δ GalNFS1* mutant cells. *β*‐galactosidase activities of cells from log‐phase cultures were assayed and normalized by that of the WT, which was arbitrarily defined as onefold. The average of three independent transformants is represented with the standard deviation. The difference between WT and *aft1Δ* is statistically significant with a *P‐*value of 0.033 based on two‐sample *t*‐test.

In an effort to establish a model system for studying the effects of iron deprivation on the human cell cycle (Fu and Richardson 2007), we used the yeast *S. cerevisiae* because of the wealth of knowledge about its genome, gene expression, and cell cycle regulation. We grew yeast under iron deprivation conditions and then analyzed the cell cycle by flow cytometry and budding index. We used DY150‐6, a *fet3‐1* mutant strain, which is defective in the major high‐affinity ferrous transporter (Askwith et al. 1994), and chelated iron in the medium (1–2 *μ*mol/L) with 100 *μ*mol/L BPS. Cells were grown to log phase in supplemented minimal SD medium and then BPS was added, which is more than sufficient to chelate most of the iron for 9 and 18 h. The cells are arrested in G1 phase of the cell cycle because they were unbudded, contained a G1 content of DNA, and continued to increase in size from 92 to 508 fL (Fig. 1) (Lew et al. 1997). Both the S‐phase CDK cyclin Clb5 and the M‐phase cyclin Clb2 are reduced in the BPS‐treated cells (Fig. 2), which is consistent with a late G1 phase arrest before production of these two cyclins (Lew et al. 1997). The arrested cells are not similar to small, nutrient‐deprived G1 cells as they continue to synthesize macromolecules and grow in size.

### Iron deprivation affects transcript levels of many iron‐regulated genes

To determine the overall transcriptome changes in cells under iron starvation, we isolated RNAs from the *fet3‐1* strain after 18 h of growth in BPS and analyzed them by microarray hybridization (Table S1). We used a statistical analysis based on *t*‐values and the FDR for the top 178 genes affected (Table 2; Table S1). At a FDR of 0.006, we would expect only one (0.006 × 178 = 1.068) to be wrong just by chance. Therefore, transcript levels of these 178 genes are significantly altered with 86 genes upregulated and 92 genes downregulated (Table 2). Many of the genes were similar to those found in other studies (Shakoury‐Elizeh et al. 2004) using wild‐type yeast strains grown under low‐iron conditions (20 *μ*mol/L Fe in media) that are less stringent than ours. Among them are genes involved in iron uptake or trafficking (*FIT1‐3*,* ARN2*,* PCA1*, and *CCC1*), oxidative stress response (*HMX1*,* GRX7*, and *CCP1*), as well as remodeling of iron utilization (*TIS11/CTH2*), all of which are part of the Aft1/2 iron regulon and are known to bind iron or are regulated by iron. Some are required for the mitochondrial ISC biogenesis pathway: *ISU2*,* FRE6*,* IBA57*, and *RIP1* (Table 2). Iron starvation is known to compromise iron–sulfur (Fe‐S) biogenesis and availability of Fe‐S clusters, which has been shown to be a key iron signal sensed by the Aft2 transcription factor (Poor et al. 2014).

**Table 2:** Summary of 178 yeast genes with transcript levels affected at least twofold in BPS

Gene name	ORF name	Cellular and molecular function	GO	Fold change in BPS
Genes induced				
*LSO1*	YJR005C‐A	Putative protein of unknown function, transcription increases during treatment with 2‐(6‐benzyl‐2‐pyridyl)quinazoline (BPQ) and copper, and is regulated by Aft1p	Molecular function unknown	220
*FIT1*	YDR534C	Mannoprotein involved in the retention of siderophore iron in the cell wall	Siderophore transport	82
*TIS11*	YLR136C	mRNA‐binding protein expressed during iron starvation	Iron homeostasis	25
*FIT3*	YOR383C	Mannoprotein involved in the retention of siderophore iron in the cell wall	Siderophore transport	20
*FIT2*	YOR383C	Mannoprotein involved in the retention of siderophore iron in the cell wall	Siderophore transport	15
*ARN2*	YHL047C	Transporter; member of the ARN family of transporters that specifically recognize siderophore iron chelates	Iron homeostasis	12
*PCA1*	YBR295W	Cadmium transporting P‐type ATPase; may also have a role in copper and iron homeostasis	Iron homeostasis	4
*ISU2*	YOR226C	Protein required for synthesis of iron–sulfur proteins; localized to the mitochondrial matrix; performs a scaffolding function in mitochondria during Fe/S cluster assembly	Iron homeostasis	3
*FRE6*	YLL051C	Protein required for synthesis of iron–sulfur proteins; localized to the mitochondrial matrix; performs a scaffolding function in mitochondria during Fe/S cluster assembly	Intracellular iron retention	3
*HMX1*	YLR205C	ER localized heme oxygenase; involved in heme degradation during iron starvation and in the oxidative stress response; expression is regulated by AFT1 and oxidative stress;	Iron homeostasis	3
*DNA2*	YHR164C	Tripartite DNA replication factor; has single‐stranded DNA‐dependent ATPase, ATP‐dependent nuclease, and helicase activities; iron–sulfur cluster binding	5′ flap endonuclease activity	2
*GRX7*	YBR014C	*Cis*‐golgi localized monothiol glutaredoxin; more similar in activity to dithiol than other monothiol glutaredoxins; involved in the oxidative stress response; iron–sulfur cluster binding	Adaptive response to oxidative stress	2
Genes repressed				
*IBA57*	YJR122W	Protein involved in incorporating iron–sulfur clusters into proteins; mitochondrial matrix protein; involved in the incorporation of iron–sulfur clusters into mitochondrial aconitase‐type proteins	Iron–sulfur cluster biosynthesis	0.5
*LIP5*	YOR196C	Protein involved in biosynthesis of the coenzyme lipoic acid; has similarity to *Escherichia coli* lipoic acid synthase; iron–sulfur cluster binding	Lipoyl synthase activity	0.3
*CCC1*	YLR220W	Vacuolar Fe^2+^/Mn^2+^ transporter; suppresses respiratory deficit of yfh1 mutants, which lack the ortholog of mammalian frataxin, by preventing mitochondrial iron accumulation; relative distribution to the vacuole decreases upon DNA replication stress	Ferrous ion transport	0.3
*GLT1*	YDL171C	NAD(+)‐dependent glutamate synthase (GOGAT); synthesizes glutamate from glutamine and *α*‐ketoglutarate; with Gln1p, forms the secondary pathway for glutamate biosynthesis from ammonia; iron–sulfur cluster binding	l‐glutamate synthase activity	0.2
*MET5*	YJR137C	Sulfite reductase beta subunit; involved in amino acid biosynthesis, transcription repressed by methionine	Cysteine synthetase activity	0.16
*GDS1*	YOR355W	Protein of unknown function; required for growth on glycerol as a carbon source; the authentic, nontagged protein is detected in highly purified mitochondria in high‐throughput studies	Molecular function unknown	0.14
*RIP1*	YEL024W	Ubiquinol cytochrome *c* reductase; a Rieske iron–sulfur protein of the mitochondrial cytochrome *bc*1 complex	Molecular function unknown	0.14
*CCP1*	YKR066C	Mitochondrial cytochrome *c* peroxidase; degrades reactive oxygen species in mitochondria, involved in the response to oxidative stress	Ferrocytochrome:hydrogen peroxide oxidoreductase activity	0.11
*LEU1*	YGL009C	Isopropylmalate isomerase; catalyzes the second step in the leucine biosynthesis pathway; iron–sulfur cluster binding	Isopropylmalate isomerase activity	0.1
*CYT1*	YOR065W	Cytochrome *c*1; component of the mitochondrial respiratory chain; expression is regulated by the heme‐activated, glucose‐repressed Hap2p/3p/4p/5p CCAAT‐binding complex	Mitochondrial electron transporter	0.1
*CYC1*	YJR048W	Cytochrome *c*, isoform 1; also known as iso‐1‐cytochrome *c*; electron carrier of the mitochondrial intermembrane space that transfers electrons from ubiquinone cytochrome *c* oxidoreductase to cytochrome *c* oxidase during cellular respiration	Mitochondrial electron transporter	0.05

BPS, bathophenanthrolinedisulfonic acid; ORF, open reading frame.

The *LSO1* gene (YJR005C‐A) was not found in the initial study (Shakoury‐Elizeh et al. 2004) and is the most increased by BPS with an over 200‐fold increase in the mRNA (Table 2). *LSO1* was not known during that original study because it was “*l*ate‐annotated *s*mall *o*pen reading frame” as the *LSO1* encodes a small 93 amino acid gene product not annotated in the original yeast genome project (Goffeau et al. 1996; Cliften et al. 2003; Kastenmayer et al. 2006). *LSO1* was annotated after being found by homology with the genome of the filamentous fungus *Ashbya gossypii* (Brachat et al. 2003). Recently, it has been suggested that *LSO1* is regulated by iron because *LSO1* mRNA was increased by about 18‐fold when yeast cells were treated with CuSO_4_ and BPQ, a chemical potentiator of copper ion accumulation, which results in a iron deficiency response via damage to mitochondrial ISCs (Foster et al. 2014a). Furthermore, many of the genes that were upregulated by BPS (Table 2) were also affected (*FIT1*,* ARN2*,* FIT2*,* FIT3*,* HMX1*,* TIS11*, and *ARN1*) by the BPQ‐CuSO_4_ treatment. In contrast, the paralogous *LSO2* gene (YGR169C‐A) was not induced by this regimen. Therefore, we analyzed the function of the *LSO1* and *LSO2* genes by using both molecular genetic and protein analyses.

### *LSO1* is regulated by the Aft1/2 transcription factors

There is no evidence of expression of *LSO1* and only SAGE RNA expression data for *LSO2* (Cliften et al. 2003; Kastenmayer et al. 2006). Our microarray data show both are expressed, but only *LSO1* is induced by iron deprivation with BPS (Table 2). Consistent with the expression data, the *LSO1* promoter region contains three consensus binding sequences (YRCACCCY) for the Aft1/2 transcription factors at nucleotide positions ‐254, ‐210, and ‐145 upstream of the start codon (Fig. 2A). In contrast, no Aft1/2 binding site was found in the 5′ upstream sequences of *LSO2*. To determine if *LSO1* is regulated by Aft1/2, we constructed a *LSO1*‐lacZ reporter plasmid and analyzed *β*‐galactosidase activities in wild‐type, *aft1Δ*,* GalNFS1*, and *aft1Δ GalNFS1* mutant cells grown in glucose‐containing SD medium (Fig. 2B). Expression of *LSO1‐lacZ* is reduced by about twofold in *aft1Δ* mutant relative to the wild‐type strain, indicating that Aft1 is required for *LSO1* transcription under normal growth conditions. Furthermore, *LSO1‐lacZ* is induced by almost 400‐fold in the *GalNFS1* mutant grown in glucose‐containing SD medium (Fig. 2B), which is known to activate the Aft1‐dependent iron regulon (Chen 2004; Rutherford et al. 2005). In this situation, Aft1/2 activation occurs because depletion of Nfs1 protein, the cysteine desulfurase that is an essential component of the mitochondrial ISC assembly machinery, leads to Fe‐S deficiency (Rutherford et al. 2005). Consistent with *AFT1* regulation, *LSO1‐lacZ* activation by *NFS1* depletion was also reduced by about twofold when *AFT1* is deleted (Fig. 2B).

### Lso1 protein expression is regulated by iron and Aft1/2 transcription factors

**Figure 3:**
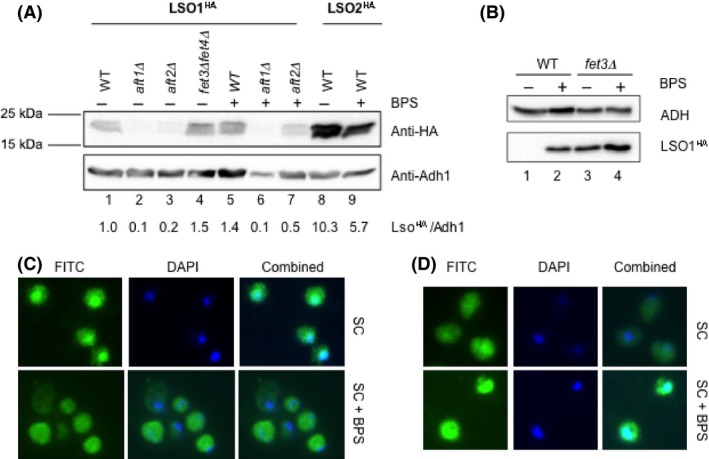
Expression of Lso1 and Lso2 proteins under iron‐replete and iron‐depleted conditions. (A) Lso1 protein expression under both iron‐replete and iron‐depleted conditions is dependent on Aft1 and Aft2, whereas Lso2 does not. WT,* aft1Δ*,* aft2Δ*, and *fet3Δ fet4Δ* cells harboring *LSO1‐3xHA* or *LSO2‐3xHA* under the control of their respective native promoters were grown in SD medium with or without 6 h incubation of 100 *μ*mol/L BPS to log phase and harvested for protein extraction. The protein blot was probed with a monoclonal anti‐HA antibody (12CA5) for Lso1^HA^ and Lso2^HA^. (B) Protein immunoblot of Lso1^HA^ of WT and *fet3Δ* mutant were grown in SD medium with or without 17 h of 200 *μ*mol/L BPS incubation. Adh1 was probed as a loading control in both (A) and (B). (C, D) Immunofluorescence staining images of a C‐terminal epitope‐tagged Lso1^HA^ (C) and Lso2^HA^ (D) in cells grown in the SC medium or SC supplemented with 100 *μ*mol/L BPS for 17 h. The combined images resulted from superimposing of 4',6‐diamidino‐2‐phenylindole (DAPI) and FITC images. WT, wild‐type; BPS, bathophenanthrolinedisulfonic acid.

To determine whether *LSO1* and *LSO2* produce protein products, the genes were tagged with a 3XHA epitope at the C‐terminus under the control of the respective native promoters. Both genes produce protein products at the expected size of 16 kDa as seen in an anti‐HA immunoblot (Fig. 3A). Lso1 protein is present in lower amounts than Lso2 protein (Fig. 3A, lanes 1 vs. 8). Similarly to *LSO1* transcription (Fig. 2B), Lso1 protein level is reduced in *aft1Δ* mutant relative to the wild‐type cells (Fig. 3A, lanes 1–2). Consistent with the observed *LSO1* transcriptional induction, Lso1 protein is induced by iron deprivation that result either from removal of both the high‐ and low‐affinity transporters in *fet3Δ fet4Δ* double mutant or from iron chelation with 100 *μ*mol/L BPS after 6 h (Fig. 3A, lanes 1 vs. 4 and 5). Moreover, *AFT1* appears to play a primary role in controlling Lso1 protein expression as Lso1 protein levels are significantly more reduced in *aft1Δ* than in *aft2Δ* mutants under growth conditions both without and with BPS chelation (Fig. 3A, lanes 1–3 and 4–7). Los1 protein is induced to higher levels if BPS treatment is increased to 200 *μ*mol/L for 17 h (Fig. 3B, lanes 1 vs. 2). Los1 expression is increased without BPS treatment in a *fet3Δ* mutant and increases further when BPS is added (Fig. 3B, lanes 3–4). This is expected as loss of the Fet3 high‐affinity iron transporter mimics iron deprivation and shows that *fet3Δ* mutant cells are essentially starved for iron (Askwith et al. 1994). We determined the subcellular localization patterns of the Lso1 protein by indirect immunofluorescence. Under iron‐replete conditions, Lso1 was enriched in the nucleus (Fig. 3C, top panel). When cells were starved for iron, both Lso1 and Lso 2 proteins appeared to be more ubiquitously distributed between the nucleus and the cytoplasm (Fig. 3C and D, bottom panel). Because the tagged Lso1 and Lso2 proteins are both regulated by iron and by the AFT1/2 transcription factors (Fig. 3), we believe they are functional, even though we do not have any direct evidence for it.

### *lso1Δ* and *lso2Δ* mutants are sensitive to iron deprivation

**Figure 4:**
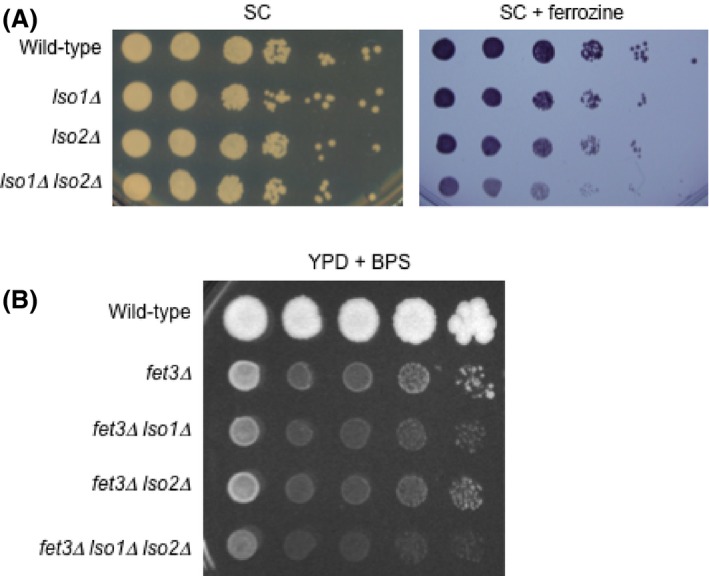
*LSO1* and *LSO2* are required for optimal growth under iron‐deficient conditions. Ten‐fold serial dilution of cells from log‐phase cultures was dot‐plated and images were taken after 3 days at 30°C. (A) Growth defect of *lso1Δ lso2Δ* double mutant under iron starvation. Congenic wild‐type, *lso1Δ*,* lso2Δ*, and *lso1Δ lso2Δ* double mutant cells were grown on SD medium supplemented with 1 mmol/L of ferrozine and 25 *μ*mol/L of ferrous iron. (B) Synthetic growth defect between *fet3Δ* and *lso1Δ lso2Δ*. Congenic wild‐type, *fet3Δ*,* fet3Δ lso1Δ*,* fet3Δ lso2Δ*, and *fet3Δ lso1Δ lso2Δ* mutant cells were on YPD medium supplemented with 100 *μ*mol/L bathophenanthrolinedisulfonic acid.

To determine if the *LSO1* and *LSO2* genes are important for the response to iron deprivation, we deleted the two genes individually and in combination. Both *lso1Δ* and *lso2Δ* single mutants are sensitive to reduced iron levels in the media that is produced using either ferrozine or BPS iron chelators. With 1 mmol/L ferrozine (having the capacity of chelating 333 *μ*mol/L Fe) and 25 *μ*mol/L of ferrous iron added to the supplemented SC medium, both *lso1*Δ and *lso2*Δ single mutants exhibit a mild slow‐growth phenotype, while the *lso1Δ lso2Δ* double mutant has an increased sensitivity (Fig. 4A). To observe sensitivity to BPS in YPD medium, we had to delete the *FET3* gene (Fig. 4B). In this case, the *lso1Δ* mutant is more sensitive than the *lso2Δ* mutant while an increased sensitivity is again seen in the *lso1Δ lso2Δ* double mutant, although not as much as with ferrozine in the *FET3* wild‐type background (Fig. 4A). As is the case on SC medium, all strains grow similarly on normal YPD medium without BPS (data not shown). From these data, we conclude that Lso1 protein plays a more important role than the Lso2 protein in response to iron deprivation, but there is a partial overlap in function of the two genes. Consistent with this notion, the Lso1 protein is induced during iron deprivation and is part of the Aft1/2 regulon.

## Discussion

Our work was the first demonstration that small open reading frames (sORFS) of yeast encode a protein product and have a cellular function. These sORFs were not annotated in the original genomic sequencing efforts because only ORFs > 100 codons were analyzed (Goffeau et al. 1996). As pointed out, there are many proteins (<100) residues that are important for cellular function, but are difficult to find from genomic data due to statistical noise resulting in the fortuitous identification of runs of in‐frame codons (Kastenmayer et al. 2006). Although many sORFS were analyzed in a genome‐wide screen, neither *LSO1* nor *LSO2* were shown to encode a protein product nor did gene knockouts have a phenotype.

Our results support the conclusion from a recent study showing that the antifungal compound copper‐BPQ disrupts iron homeostasis because expression of many of the same genes affected by copper‐BPQ (Foster et al. 2014b) is also affected by direct iron chelation with BPS (Table 2). In both studies, the *LSO1* gene was found to be a target of iron deprivation, unlike the paralogous *LSO2* gene. Thus, we also conclude that copper‐BPQ mimics the effect of iron deprivation and induces iron‐regulon pathway via the Atf1/2 transcription factors.

**Figure 5:**
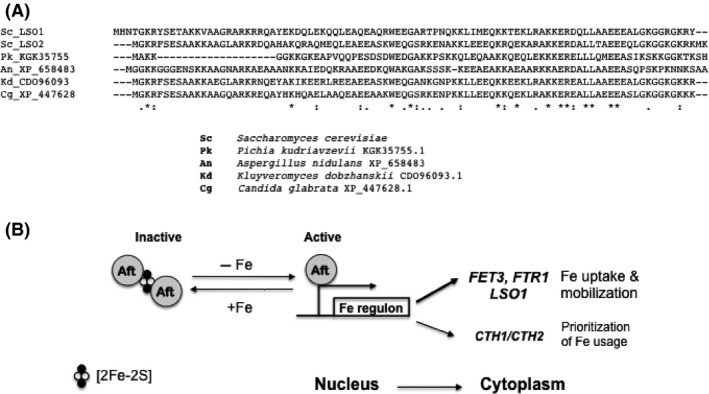
A model for LSO1 being part of the Aft1/2‐regulated iron regulon in yeast. (A) Alignment of fungal Lso1 and Lso2 proteins from different fungi. (B) Iron depletion leads to an increase of the active (monomeric) form of the Aft1/2 transcription factors, which binds to target promoters of many genes of the iron regulon to activate transcription. The products of these genes including *LSO1*, produce a myriad of functions important for the response to iron deprivation.

Based on the evidence in this and the previous report (Foster et al. 2014a), it is reasonable to propose the Lso1 and Lso2 proteins are involved in iron homeostasis. The *LSO2* gene is constitutively expressed and may provide a low level of response, while higher levels of the Lso1 protein may be needed for the full response under low‐iron conditions and hence it is inducible. These two paralogous proteins are found in many fungi (Fig. 5A) with some being full length and others sharing homology only at their C terminal halves. Lso1 and Lso2 homologs are present mainly in the large fungal phylum Ascomycota (Fig. 5A) and not in other fungi. Perhaps they have evolved to adapt to the limiting iron conditions in the environment of these organisms (Philpott and Protchenko 2008; Outten and Albetel 2013). Indeed, iron regulation by the ATF1/2 regulon is important in virulence of the pathogenic yeast *Candida albicans* (Liang et al. 2010). Coiled coil domains are predicted for Lso1 residues 20–41 and 59–83, and for Lso2 residues 17–38 and 47–80, these domains are likely involved in protein–protein interactions.

Aft1‐controlled genes are more likely involved in iron transport at the cell membrane, while Aft2‐controlled genes are in intracellular iron trafficking (Courel et al. 2005). Because deletion of *AFT1* has a more dramatic effect on *LSO1* basal and inducted expression and deletion of the Fet3 iron transporter increases Lso1 protein levels (Fig. 3), Lso1 protein may be involved in iron transport at the cell membrane (Fig. 5B). This is consistent with the increased cytoplasmic localization of Lso1 observed during iron deprivation (Fig. 3C). The paralog *LSO2* is not regulated by Fe. Neither is the *LSO1* adjacent gene *POL31*. Consistent with the transcriptional induction of *LSO1* by iron deprivation, the *LSO1* promoter region contains three consensus Aft1/2 binding sites YRCACCCR (Fig. 2A), while *LSO2* promoter has none. The best fit to the consensus is site A2 with 8/8 matches. These sites are conserved in many *Saccharomyces* strains and species. Even in the distantly related filamentous fungus *A. gossypii LSO1* homolog AAL130W, there is an Aft site (GCACCCA) in a similar location about 114–120 bp upstream of the initiator ATG.

We found that iron deprivation leads to a pronounced cell cycle arrest after about three generations in BPS (Fig. 1). Defects in cell cycle‐specific events lead to accumulation of yeast cells at a specific point in the cell cycle (Hartwell 1974). We have found that yeast cells depleted of iron arrest in G1 of the cell cycle at a point similar to START in that the cells are large and unbudded and arrested before production of the S‐phase cyclin Clb5 and the M‐phase cyclin Clb2 (Lew et al. 1997) (Fig. 1). These results are similar to that found in human cancer cells, which also arrest in G1 phase when treated with an iron chelator (Brodie et al. 1993). These human cancer cells arrest after production of the G1/S‐phase cyclin E, but before production of the S‐phase cyclin A (Siriwardana and Seligman 2013). The human cancer cells grown under iron starvation also exhibited a second arrest point in S phase as a result of the inhibition of RNR (ribonucleotide reductase), which is needed to produce deoxynucleotides (Siriwardana and Seligman 2013). We did not detect this second point in yeast perhaps because the first point in G1 phase is more sensitive to the effect of iron depletion than the latter point in S phase. We propose our results show yeast is a valuable model for studying the effect of iron deprivation on the cell cycle in human cancer cells.

## Conflict of Interest

None declared.

## Supporting information


**Table S1.** Complete microarray data for RNA of 178 yeast genes with transcript levels affected at least twofold in BPS.
